# Hemoglobin levels and clinical outcomes after extracorporeal circulation auxiliary to open heart surgery: a retrospective cohort study

**DOI:** 10.1186/s12872-023-03647-4

**Published:** 2023-12-07

**Authors:** Zhao-kun Fan, Zhi-rong Zhang, Ru-qin Yi, Wen Feng, Cheng-en Li, Wei Chen, Ying-ying Shen

**Affiliations:** 1 Department of Intensive Care Unit, the First Affiliated Hospital of Zhejiang Chinese Medical University (Zhejiang Provincial Hospital of Chinese Medicine), Hangzhou, 310006 China; 2 Department of Medical Record, the First Affiliated Hospital of Zhejiang Chinese Medical University (Zhejiang Provincial Hospital of Chinese Medicine), Hangzhou, 310006 China

**Keywords:** Hemoglobin, Extracorporeal circulation, Mortality, ICU

## Abstract

**Background:**

Extracorporeal circulation auxiliary to open heart surgery is a common procedure used to treat heart diseases. However, the optimal transfusion strategy for patients undergoing this surgery remains a subject of debate. This study aims to investigate the association between hemoglobin levels and clinical outcomes in patients undergoing extracorporeal circulation auxiliary to open heart surgery, with the ultimate goal of improving surgical success rates and enhancing patients' quality of life.

**Methods:**

A retrospective analysis was conducted on data from the Medical Information Mart for Intensive Care IV 2.2 (MIMIC-IV 2.2) database, including 4144 patients. The patients were categorized into five groups based on their minimum hemoglobin levels during hospitalization. Baseline characteristics, clinical scores, laboratory results, and clinical outcome data were collected. Statistical analyses utilized descriptive statistics, ANOVA or Kruskal-Wallis tests, Kaplan-Meier method, and Log-rank test.

**Results:**

The results revealed a significant correlation between hemoglobin levels and in-hospital mortality, as well as mortality rates at 30 days, 60 days, and 180 days (*p* < 0.001). Patients with lower hemoglobin levels exhibited higher mortality rates. However, once hemoglobin levels exceeded 7g/dL, no significant difference in mortality rates was observed (p = 0.557). Additionally, lower hemoglobin levels were associated with prolonged hospital stay, ICU admission time, and mechanical ventilation time (*p* < 0.001). Furthermore, hemoglobin levels were significantly correlated with complication risk, norepinephrine dosage, and red blood cell transfusion volume (*p* < 0.001). However, there was no significant difference among the groups in terms of major complications, specifically sepsis (p > 0.05).

**Conclusion:**

The study highlights the importance of managing hemoglobin levels in patients undergoing heart surgery with extracorporeal circulation. Hemoglobin levels can serve as valuable indicators for predicting clinical outcomes and guiding treatment decisions. Physicians should carefully consider hemoglobin levels to optimize transfusion strategies and improve postoperative patient outcomes. Further research and intervention studies are warranted to validate and implement these findings in clinical practice.

## Introduction

The transfusion therapy strategy for critically ill patients has long been a central issue in clinical decision-making [1–3]. Hemoglobin, as the primary carrier of oxygen, plays a crucial role in maintaining tissue oxygen supply [4]. Consequently, low hemoglobin levels may affect oxygen delivery, leading to organ dysfunction or even organ failure [5–7]. Most current clinical guidelines recommend considering transfusion when hemoglobin levels in critically ill patients drop to 7 g/dL or below [8]. This recommendation is based on numerous studies involving patients in various clinical scenarios [9]. For example, certain studies focusing on different clinical contexts have found that hemoglobin levels below specific thresholds may be associated with adverse outcomes [10]. However, on the other hand, excessive transfusion may also lead to other complications, such as transfusion-related acute lung injury, infections, and other transfusion-related reactions [11]. Nevertheless, whether this recommendation still applies to patients undergoing extracorporeal circulation auxiliary to open heart surgery remains a question that needs to be explored. The heart plays a critical role in the oxygen delivery system of the human body [12]. For patients undergoing extracorporeal circulation auxiliary to open heart surgery(EC-AOHS ), the invasiveness of the procedure and associated systemic stress responses may affect their oxygen delivery and utilization. Additionally, EC-AOHS patients often have various underlying diseases, which may further increase their risk of developing anemia. The goal of red blood cell transfusion is to improve or maintain oxygen delivery in patients with hemorrhage or anemia [13]. The decision for transfusion becomes complex due to multiple factors; severe anemia and excessive blood loss are common, and cardiovascular disease patients may have different transfusion requirements compared to other patient populations. The decision for transfusion in cardiac surgery is often based on the severity of perioperative anemia. Guidelines (Grade C recommendation) suggest a highly restrictive transfusion threshold with a hemoglobin concentration of 60–70 g/L [8]. These thresholds are mainly derived from randomized controlled trials in non-cardiac surgery patients, which have demonstrated the equivalence of restrictive transfusion thresholds [14]. They have also drawn evidence from observational studies, which show that reversing severe anemia with red blood cell transfusions is associated with adverse clinical outcomes such as death, acute lung injury, acute kidney injury, stroke, myocardial infarction, sepsis, and surgical site infection [15]. Red blood cell transfusion carries significant morbidity. Hemolytic transfusion reactions and transfusion-related lung injury cause many deaths each year, but the causal relationship between red blood cell transfusions and observed adverse outcomes remains undetermined [16]. Severe anemia is the primary indication for transfusion and an important predictor of adverse outcomes in patients undergoing cardiac surgery, who may already be at the limits of their physiological reserve. Therefore, for EC-AOHS patients, a separate evaluation of the relationship between hemoglobin levels and clinical outcomes is needed to determine the optimal transfusion strategy.

We used the MIMIC-IV database to investigate the relationship between hemoglobin levels and clinical outcomes in EC-AOHS patients. The MIMIC-IV database is a large, publicly available critical care database that contains extensive clinical data from various healthcare institutions. Analyzing this data allows us to study a large number of patients efficiently and draw reliable conclusions. Such research is valuable for optimizing treatment strategies and improving postoperative outcomes for EC-AOHS patients.

## Method

### Data source

We conducted a large sample retrospective analysis using the MIMIC-IV database (version 2.2) [17]. This database is developed by the computational physiology laboratory of the Massachusetts Institute of Technology (MIT, Cambridge, MA, USA) with approval from the Institutional Review Boards (IRB) of MIT and Beth Israel Deaconess Medical Center (BIDMC), contains demographic data, vital signs, laboratory results, and prescriptions collected between 2008 and 2019 in the ICU or emergency department of BIDMC. Patient identifications were removed to comply with the Health Insurance Portability and Accountability Act’s (HIPAA) Safe Harbor provision [18, 19]. One author, Zhao kun Fan, completed the online course on Data or Specimens Only Research and gained access to the database for data extraction after passing the Collaborative Institutional Training Initiative (CITI) exam (Record ID: 55,303,422).

### Study population and data collection

The study cohort consisted of 4,144 patients with extracorporeal circulation auxiliary to open heart surgery from the MIMIC-IV, who were identified based on the Ninth (ICD-9) and Tenth (ICD-10) Revision of Interna tional Classification of Diseases. We included patients who underwent extracorporeal circulation auxiliary to open heart surgery. Based on the minimum hemoglobin value during the hospitalization, patients were divided into five groups: Group 1 (≤ 7.0 g/dL), Group 2 (7.1-8.0 g/dL), Group 3 (8.1-9.0 g/dL), Group 4 (9.1–10.0 g/dL), and Group 5 (> 10.0 g/dL).

**Fig. 1:**
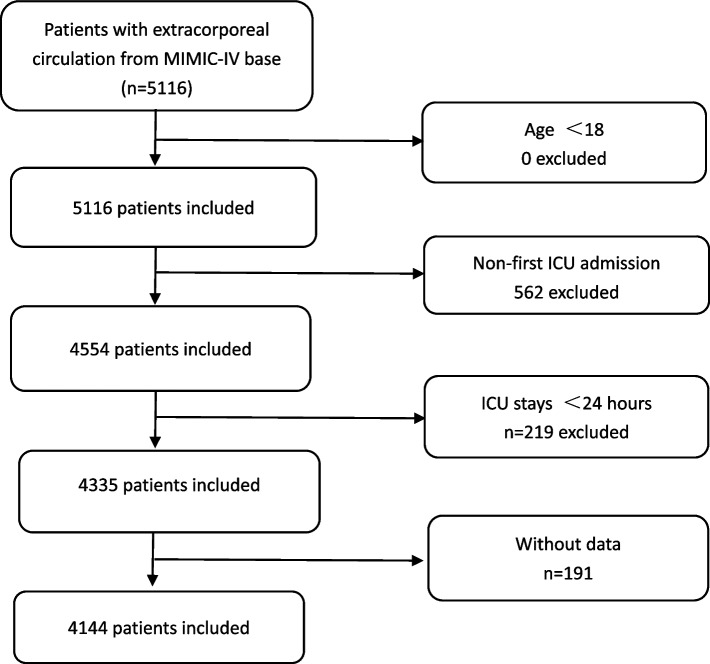
Flowchart of subject screening

Patients with one of the following conditions were excluded: (1) lacked hemoglobin data; (2) less than 18 years old; (3) patients with repeated ICU admissions (If a patient was admitted to the ICU more than once, we used the date of the first admission); (4) less than 24 h of ICU stay; The detailed inclusion of our enrolled patients is shown in Fig. 1.

Data collection included the following: demographic data, comorbidities, vital signs, laboratory parameters, medication use, length of hospital stay, length of ICU stay (ICU LOS), and time of death. These data were collected using a PostgreSQL database.

1. Demographic data includes age, gender.
2. Complications include diabetes, hypertension, coronary heart disease (CAD), congestive heart failure (CHF), chronic kidney disease (CKD) and chronic obstructive pulmonary disease (COPD), tumors.
3. Vital signs include respiratory rate (RR), heart rate (HR), mean blood pressure (MBP) and transcutaneous oxygen saturation (SpO2).
4. Laboratory indicators include white blood cell count (WBC), red blood cell count (RBC), minimum value of hemoglobin (hemoglobin min) during admission, platelet count (PLT), international normalized ratio (INR), activated partial thromboplastin time ( PTT).
5. The scoring systems included the Sequential Organ Failure Assessment score (SOFA), the Oxford Acute Severity of Illness Score (OASIS), and the Acute Physiology Score III (APS III).
6. Treatment information included the dose of norepinephrine used, the amount of red blood cells transfused, and the duration of mechanical ventilation.
7. Additionally, the study recorded the length of hospital stay, ICU length of stay , and in-hospital, 30-day, 60-day, and 180-day all-cause mortality.

#### Statistical analysis

Descriptive statistics were used to summarize the baseline characteristics and clinical outcomes of the patients. The distribution differences of continuous variables among the groups were compared using ANOVA or Kruskal-Wallis test, while categorical variables were compared using Chi-squared or Fisher’s exact test. Survival analysis was performed using the Kaplan-Meier method, and differences among the groups were compared using the Log-rank test.

### Outcomes

#### Definitions

The baseline hemoglobin is defined as the first recorded data upon admission, while the minimum hemoglobin represents the lowest value detected during the hospital stay. The hemoglobin change is calculated as the difference between the baseline value and the minimum value. Patients were divided into five groups: Group 1 (≤ 7.0 g/dL), Group 2 (7.1-8.0 g/dL), Group 3 (8.1-9.0 g/dL), Group 4 (9.1–10.0 g/dL), and Group 5 (> 10.0 g/dL).

### Statistical analysis

Continuous variables were represented as mean ± standard deviation (SD) for data adhering to normal distribution and as median (25th, 75th percentiles) for non-normally distributed data. The analytical tools employed were one-way ANOVA and Kruskal-Wallis H-test, respectively. Categorical data were denoted as frequency (percentage) and assessed via Chi-square or, when applicable, Fisher’s exact tests. We presumed missing data to occur at random and thus employed multiple imputations for completion.

Survival distributions, contingent on different hemoglobin levels, were juxtaposed using the Kaplan-Meier method, accompanied by the log-rank test. The results were elucidated as cumulative incidence.

### For a more intuitive understanding, we incorporated several visualization methodologies

Violin Plots were deployed for a granular understanding of hospital durations, encompassing metrics like the overall stay, ICU stay, and mechanical ventilation duration.

Box Plots were pivotal in extrapolating treatment specifics, with a keen focus on red blood cell transfusion quantities and the norepinephrine administration frequency.

Heatmaps vividly encapsulated the sepsis incidence data.

To further probe the interdependencies among variables, we leveraged Correlation Tools. The synergy of heatmaps and matrix plots was especially informative, casting light on correlations involving age, gender, in-hospital mortality, and diverse scores.

All statistical inferences were drawn using STATA (version 17.0), with the significance threshold earmarked at *p* < 0.05.

## Results

### Patient characteristics

This study enrolled a total of 4,144 patients who were stratified into five groups based on their minimum hemoglobin levels during hospitalization. The distribution of patients in each group was as follows: 381 patients in the group with hemoglobin ≤7.0 g/dL, 1,145 patients in the group with 7.1-8.0 g/dL, 1,312 patients in the group with 8.1-9.0 g/dL, 801 patients in the group with 9.1-10.0 g/dL, and 505 patients in the group with hemoglobin >10.0 g/dL. The baseline characteristics of these patients are presented in Table 1. Age: The average age across all groups is 67.86 years. The group with Hb>10.0g/dl has the lowest average age, at 62.40 years, while the Hb 7.1-8.0g/dl group has the highest average age, at 69.60 years. Based on the *P*-value, there's a statistically significant difference in age distribution between different Hb level groups. Gender: Overall, 68.07% of the sample are male.In the Hb>10.0g/dl group, the proportion of males is the highest, reaching 97%.

**Table 1:** Baseline characteristics

Variables	HB(g/dl) (*n* = 4144)	Hb ≤ 7 g/dl (*n* = 381)	Hb7.1-8.0 g/dl (*n* = 1145)	Hb8.1-9.0 g/dl (*n* = 1312)	Hb9.1-10.0 g/dl (*n* = 801)	Hb (> 10.0 g/dl (*n* = 505)	*P* Value
Age (yr)	67.86(12.09)	68.36(14.55)	69.60(12.26)	69.07(11.35)	66.60(11.46)	62.40(10.67)	<0.001
Male(%)	2821(68.07)	192(50.4)	651(57)	862(66)	677(60.15)	489(97)	<0.001
OASIS Score	31.63(8.00)	33.86(8.53)	33.09(8.04)	31.82(7.76)	30.00(7.67)	28.77(7.33)	<0.001
SOFA Score	5.43(2.79)	6.03(3.38)	5.71(3.04)	5.43(2.73)	5.05(2.44)	4.96(2.17)	<0.001
APSIII Score	40.30(19.20)	44.77(20.05)	43.32(19.70)	39.96(18.74)	37.52(18.47)	35.33(17.86)	<0.001
R rate (bpm)	17.47(2.65)	17.90(3.30)	17.49(2.68)	17.37(2.65)	17.33(2.32)	17.57(2.46)	0.146
Heart rate (bpm)	82.88(9.48)	83.37(10.83)	83.05(9.49)	82.75(9.40)	82.86(9.06)	82.53(9.26)	<0.001
MAP (mmHg)	74.21(6.22)	73.65(7.11)	73.45(6.58)	73.82(6.17)	75.10(5.42)	75.97(5.44)	<0.001
SpO_2_ (%)	97.88(1.37)	98.08(1.68)	98.04(1.45)	97.94(1.31)	97.76(1.22)	97.41(1.19)	<0.001
Myocardial infarct	1064	110	312	341	196	105	0.033
Congestive Heart failure	1154	137	395	367	176	79	<0.001
Cerebrovascular disease	444	60	168	128	61	27	<0.001
Renal disease	612	83	252	192	55	30	<0.001
Chronic pulmonary disease	1033	102	345	337	171	78	<0.001
Malignant cancer	126	17	38	43	23	5	0.034
Hemoglobin min(g/dl)	8.54(1.25)	6.47(0.66)	7.61(0.28)	8.52(0.28)	9.52(0.29)	10.78(0.58)	<0.001
Hemoglobin change(g/dl)	2.62(2.18)	3.30(2.61)	2.74(2.20)	2.62(2.17)	2.39(2.02)	2.18(1.84)	<0.001
RBC min(×10^12^/L)	2.82(0.42)	3.20(0.32)	3.22(0.29)	3.35(0.31)	3.57(0.31)	3.92(0.34)	<0.001
Platelet mean(×10^9^/L)	183.88(64.67)	187.45(74.26)	191.63(75.15)	181.72(60.97)	179.98(56.23)	175.41(49.61)	0.018
WBC mean(×10^9^/L)	11.25(3.71)	11.36(5.73)	11.11(3.87)	11.05(3.14)	11.35(3.60)	11.88(2.72)	<0.001
INR mean(s)	1.39(0.34)	1.48(0.36)	1.44(0.38)	1.38(0.33)	1.33(0.28)	1.34(0.32)	<0.001
PTT mean(s)	38.12(11.36)	41.37(11.53)	39.81(12.28)	37.79(11.00)	36.37(10.60)	35.46(9.90)	<0.001

Data are presented as median (interquartile range), or number of patients(%)

*OASIS* Oxford Acute Severity of Illness Score; *SOFA* sequential organ failure assessment; *APSIII* Acute Physiology Score III; *R* rate respiration rate; *MAP* mean arterial blood pressure; *RBC* red blood cell; *WBC* white blood cell; *INR* international normalized ratio; *PTT* activated partial thromboplastin time

 Basic illness: Myocardial Infarction: 1064 cases, with the Hb8.1-9.0g/dl group having the most at 341 cases.Congestive Heart Failure: 1154 cases, with the Hb7.1-8.0g/dl group having the most at 395 cases.Cerebrovascular Disease: 444 cases, with the Hb7.1-8.0g/dl group having the most at 168 cases.Renal Disease: 612 cases, with the Hb7.1-8.0g/dl group having the most at 252 cases.Chronic Pulmonary Disease: 1033 cases, with both the Hb7.1-8.0g/dl and Hb8.1-9.0g/dl groups having 345 cases each.Malignant Cancer: 126 cases, with the Hb8.1-9.0g/dl group having the most at 43 cases.

 Other Indicators: OASIS Score: The average for all groups is 31.63. The Hb>10.0g/dl group has the lowest average score of 28.77.SOFA Score: The average for all groups is 5.43. The Hb>10.0g/dl group has the lowest average score of 4.96.APSIII Score: The average for all groups is 40.30. The Hb>10.0g/dl group has the lowest average score of 35.33.Respiratory Rate (R rate): The average for all groups is 17.47 bpm. The Hb≤7g/dl group has the highest average rate of 17.90 bpm. Heart Rate: The average for all groups is 82.88 bpm. The Hb≤7g/dl group has the highest average rate of 83.37 bpm.Mean Arterial Pressure (MAP): The average for all groups is 74.21 mmHg. The Hb>10.0g/dl group has the highest average at 75.97 mmHg.Oxygen Saturation (SpO2): The average for all groups is 97.88%. The Hb>10.0g/dl group has the lowest average at 97.41%.

 Laboratory Test Indicators: Hemoglobin (Min) (g/dl): The overall average is 8.54 (1.25). The Hb>10.0g/dl group has the highest average value of 10.78 (0.58), while the lowest is 6.47 (0.66) for the Hb≤7g/dl group.Hemoglobin Change (g/dl): The overall average change is 2.62 (2.18). The Hb≤7g/dl group had the highest change with 3.30 (2.61).RBC (Min) (×10^12/L): The overall average is 2.82 (0.42). The Hb>10.0g/dl group recorded the highest average value of 3.92 (0.34). Platelet Mean (×10^9/L): The average across all groups is 183.88 (64.67). The Hb7.1-8.0g/dl group had the highest average value of 191.63 (75.15). WBC Mean (×10^9/L): The average white blood cell count across all groups is 11.25 (3.71). The Hb>10.0g/dl group recorded the highest average value of 11.88 (2.72). INR Mean (s): The overall average is 1.39 (0.34). The Hb≤7g/dl group had the highest average value of 1.48 (0.36). PTT Mean (s): The average across all groups is 38.12 (11.36). The Hb≤7g/dl group had the highest average value of 41.37 (11.53).

### Top ten primary diagnoses

**Fig. 2:**
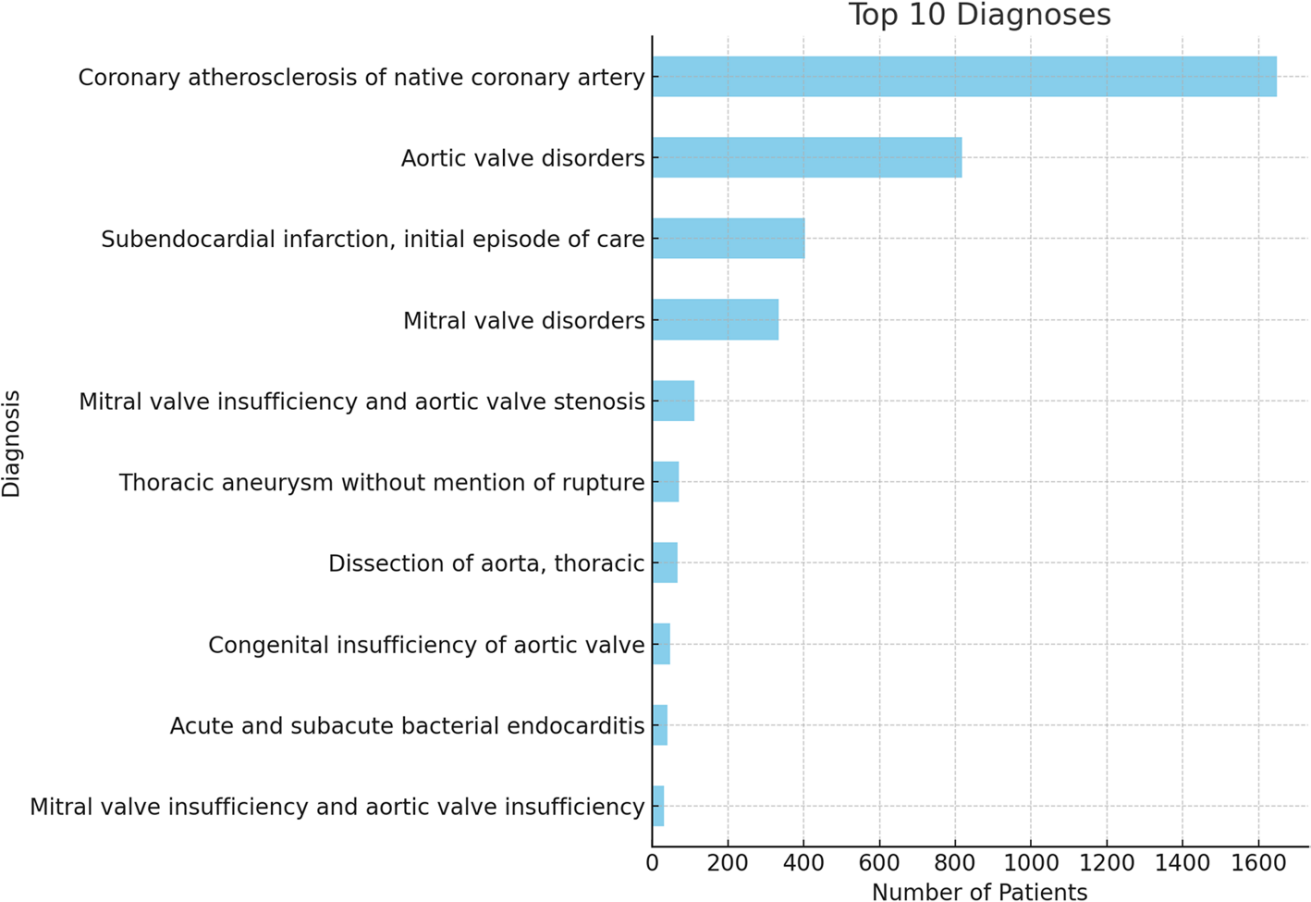
Top ten primary diagnoses

Additionally, Fig. 2 illustrates the top ten primary diagnoses observed among the study participants. From it, we can observe the frequency or count of different diagnoses. Notably, “coronary atherosclerosis of native coronary artery” has the highest number of patients, ranking it first. This indicates that within the given sample or dataset, this diagnosis is the most prevalent.

### Comparison of outcome measures

In this study, patients with different hemoglobin levels were grouped, and multiple outcome measures were compared among the groups. The outcomes are presented in Table 2.

**Table 2:** Outcomes

Variables	HB(g/dl) (*n* = 4144)	Hb ≤ 7 (*n * = 381)	Hb7.1-8.0 (*n * = 1145)	Hb8.1-9.0 (*n* = 1312)	Hb9.1-10.0 (*n* = 801)	Hb > 10.0 (*n* = 505)	*P *value
Los hospital(day)	9.07(6.82)	13.86(12.55)	10.63(7.48)	8.62(5.29)	7.09(3.57)	6.25(2.38)	*<0.001*
Los ICU(day)	3.17(4.22)	5.43(7.66)	3.88(4.96)	3.04(3.37)	2.20(2.05)	1.73(1.20)	*<0.001*
Hospital expire flag(%)	58(1.40%)	22(5.77%)	22(1.92%)	12(0.91%)	1(0.12%)	1(0.20%)	*<0.001*
Death within 30 days(%)	59(1.42%)	20(5.25%)	22(1.92%)	14(1.07%)	1(0.12%)	1(0.40%)	*<0.001*
Death within 60 days(%)	95(2.29%)	29(7.61%)	36(3.14%)	25(1.91%)	3(0.37%)	2(0.40%)	*<0.001*
Death within 180 days(%)	154(3.72%)	43(11.29%)	60(5.24%)	41(3.13%)	6(0.75%)	4(0.80%)	*<0.001*
RBC Totalamount(ml)	687.24 (1516.53)	1978.38 (3030.63)	1025.46 (1583.11)	567.49 (1109.38)	176.30 (408.63)	67.77 (684.37)	*<0.001*
Duration (hour)	49.65(65.04)	82.17(112.84)	60.65(76.79)	49.15(54.17)	34.01(32.21)	26.24(21.37)	*<0.001*
Vaso amount(ml)	2.60(16.57)	8.04(38.08)	3.59(16.26)	2.19(13.65)	0.74(5.03)	0.26(1.61)	*<0.001*
Sepsis(%)	3091	264(69.29%)	848(74.06%)	989(75.38%)	617(77.02%)	375(74.25%)	*0.067*

*LOS length of stay; VASO *Vasoactive substances

**Fig. 3:**
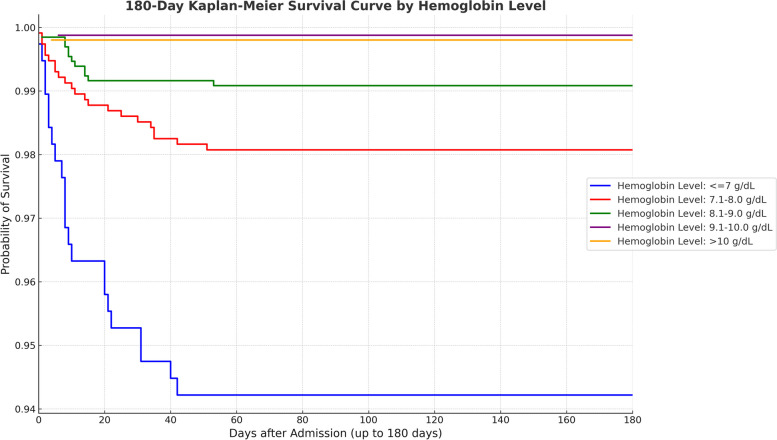
Survival analysis of 180-day kaplan-meier survival curve by hemoglobin level

Additionally, we conducted survival analysis to explore the relationship between hemoglobin levels and survival time (Fig. 3). From the Kaplan-Meier survival curves, we observed that as hemoglobin levels increased, the survival probability seemed to improve. Particularly in the group with lower hemoglobin levels, the survival rate was significantly lower than in the other groups. However, the Log-rank test revealed no significant differences in survival time within 180 days between the four groups: 7.1-8.0, 8.1-9.0, 9.1–10, and > 10 (*p*-value = 0.557).

### Visual comparison of secondary outcome measures

**Fig. 4:**
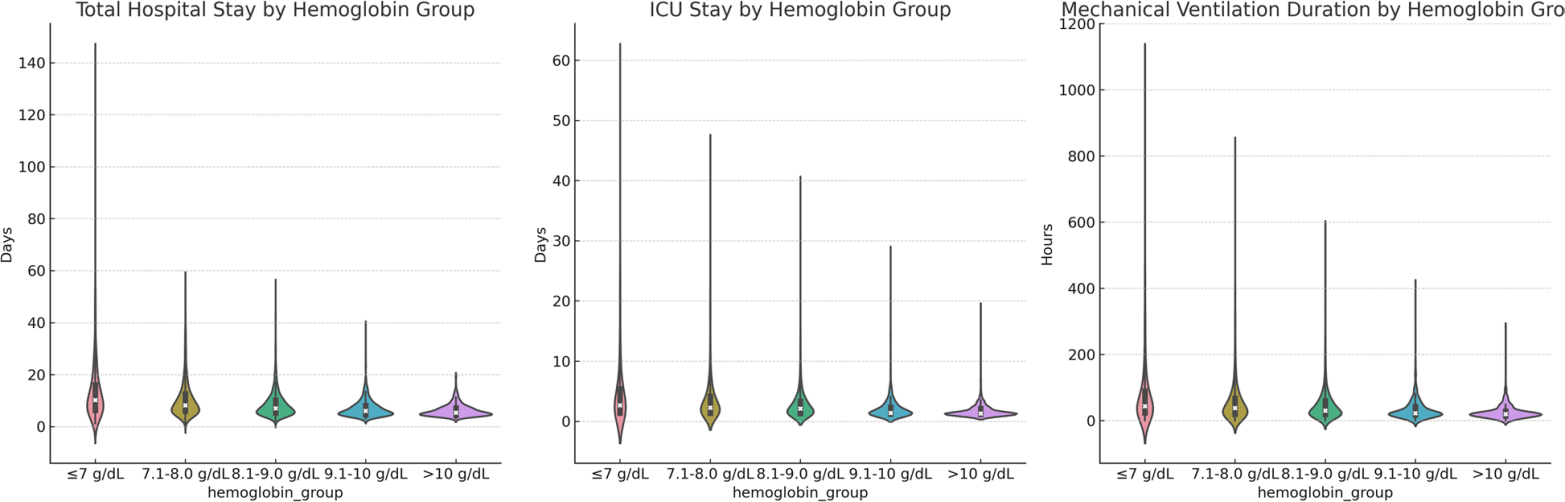
Violin plots for length of hospital stay, length of ICU stay, mechanical ventilation time

To visually display the differences in secondary outcome measures among different hemoglobin level groups, we used violin plots for visualization (Fig. 4).

#### Length of hospital stay

From the figure, it can be observed that the group with hemoglobin levels less than or equal to 7 g/dL had a wider distribution of hospital stay duration. In contrast, as hemoglobin levels increased, the median and distribution range of hospital stay duration decreased, especially in the group with hemoglobin levels greater than 10 g/dL.

#### Length of ICU stay

For ICU stay duration, the group with hemoglobin levels less than or equal to 7 g/dL had a relatively higher median. Similar to the trend in total hospital stay duration, as hemoglobin levels increased, the median and distribution range of ICU stay duration also decreased.

#### Mechanical ventilation time (duration hours)

Regarding mechanical ventilation time, the group with hemoglobin levels less than or equal to 7 g/dL had the widest distribution and a relatively higher median. However, when hemoglobin levels increased to above 10 g/dL, the median and distribution range of mechanical ventilation time significantly decreased.

Overall, lower hemoglobin levels (less than or equal to 7 g/dL) were associated with longer hospital stay, longer ICU stay, and longer mechanical ventilation time.

#### Differences between groups in other indicators

To further evaluate the differences between different hemoglobin level groups, we analyzed the amount of red blood cell transfusion, the use of norepinephrine, and the incidence of sepsis.

#### Amount of red blood cell transfusion

**Fig. 5:**
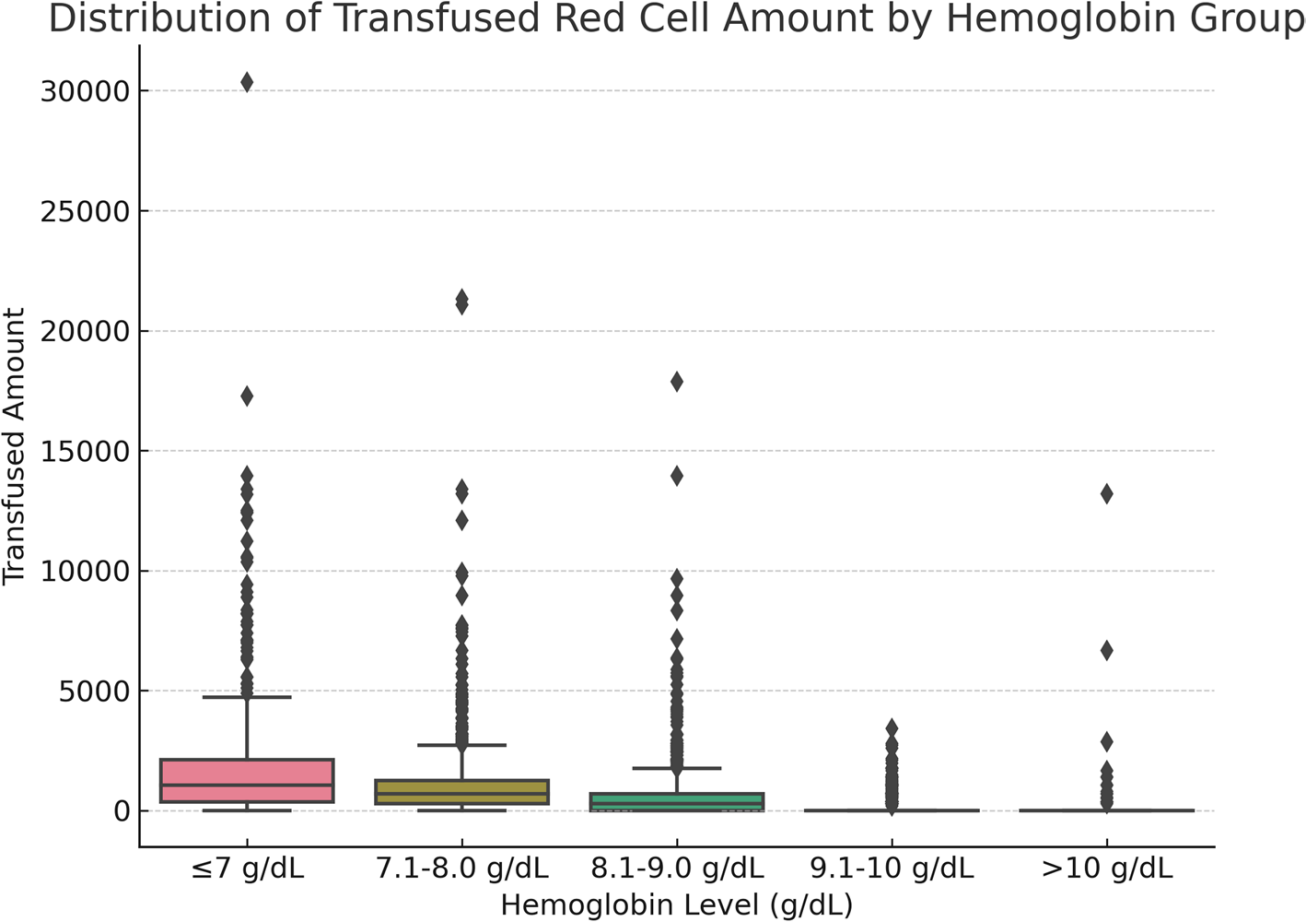
Box plots of amount of red blood cell transfusion

The box plots (Fig. 5) show that the group with hemoglobin levels less than or equal to 7 g/dL had a wide variation in the amount of red blood cell transfusion. As hemoglobin levels increased, the median and distribution range of red blood cell transfusion decreased.

#### Use of norepinephrine

**Fig. 6:**
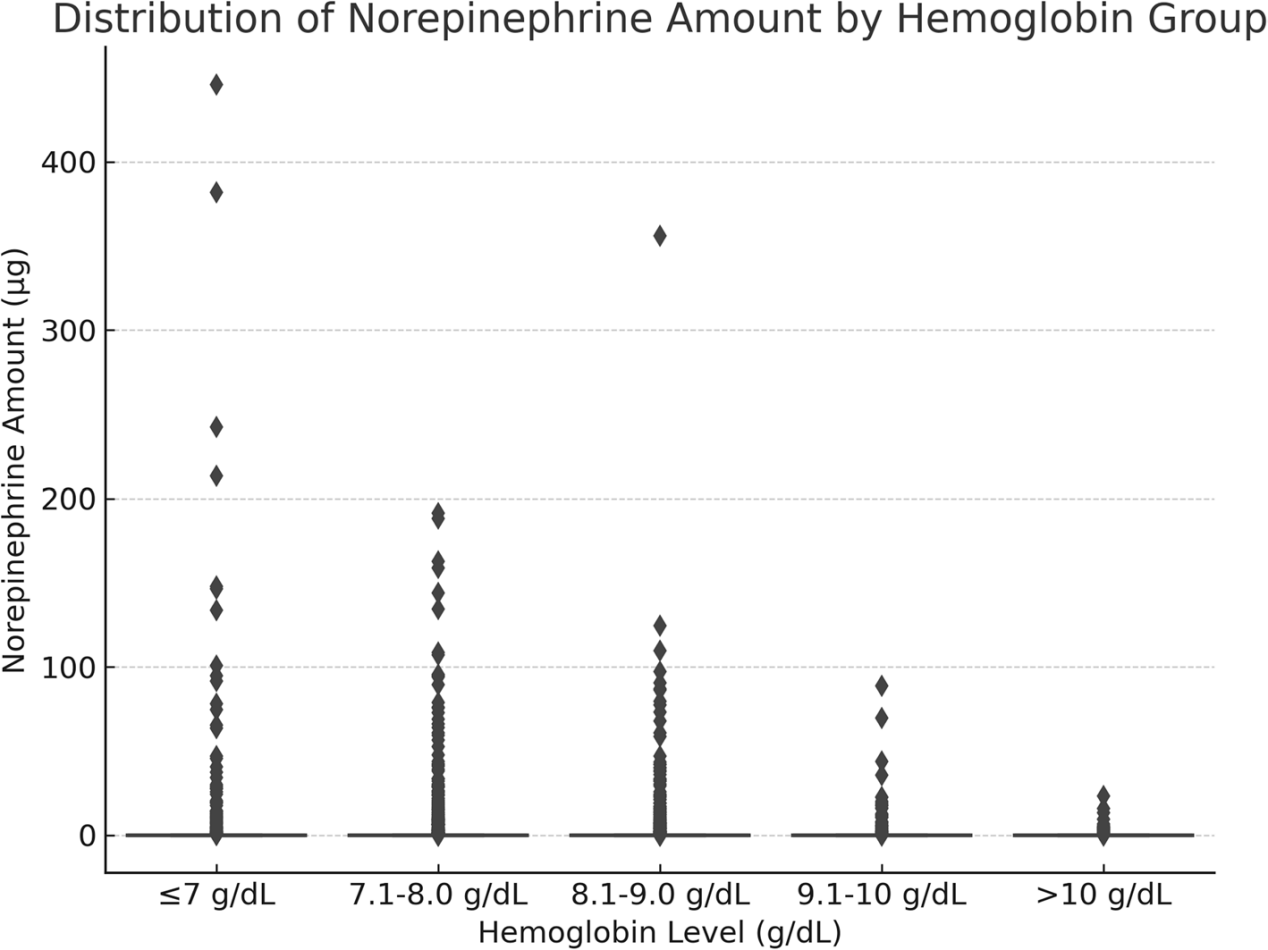
Use of norepinephrine

For the use of norepinephrine (Fig. 6), the group with hemoglobin levels less than or equal to 7 g/dL had a relatively higher median, but with greater variation. As hemoglobin levels increased, the median and distribution range of norepinephrine use decreased.

#### Incidence of sepsis

**Fig. 7:**
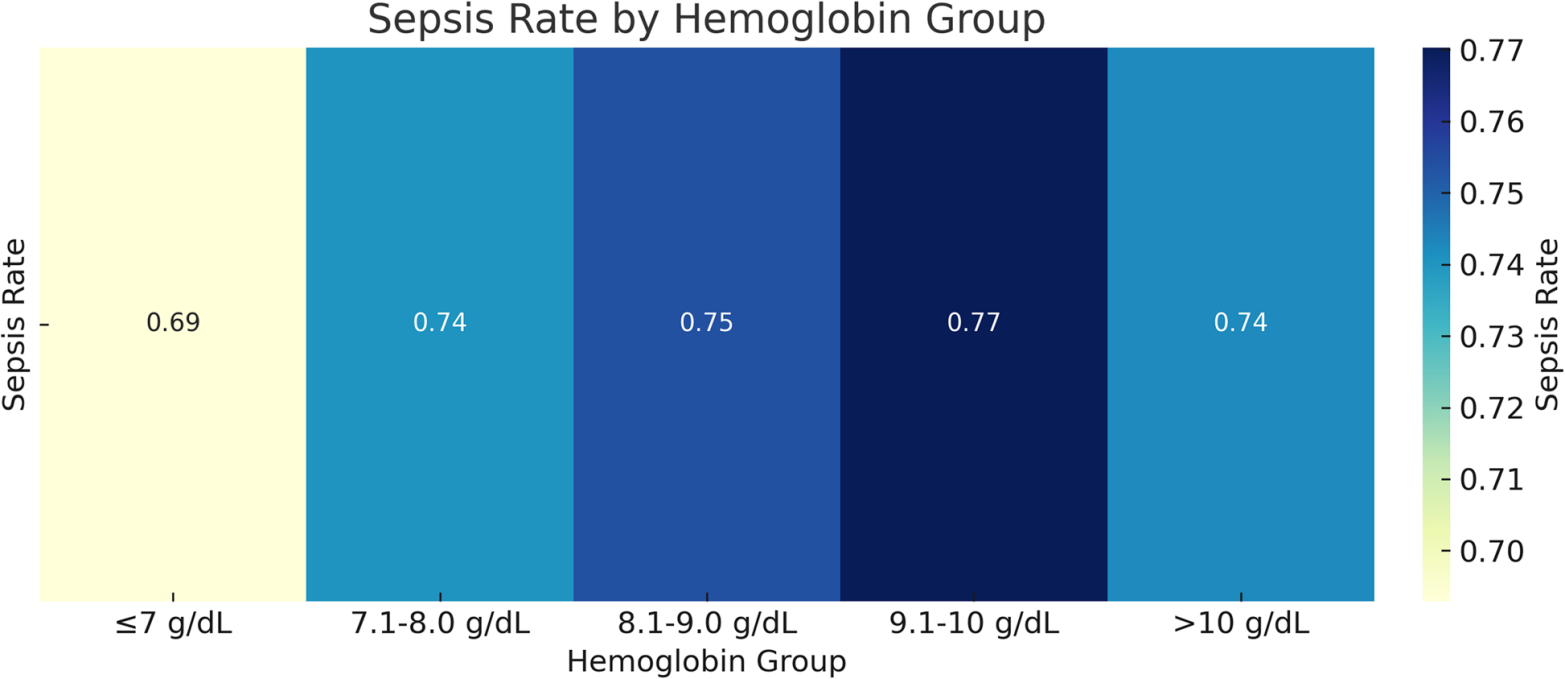
Heatmap illustrating differences in the incidence of sepsis among different hemoglobin groups

The heatmap (Fig. 7) illustrates that different hemoglobin groups had some differences in the incidence of sepsis, although these differences did not reach statistical significance.

#### Relationship analysis of relevant variables

**Fig. 8:**
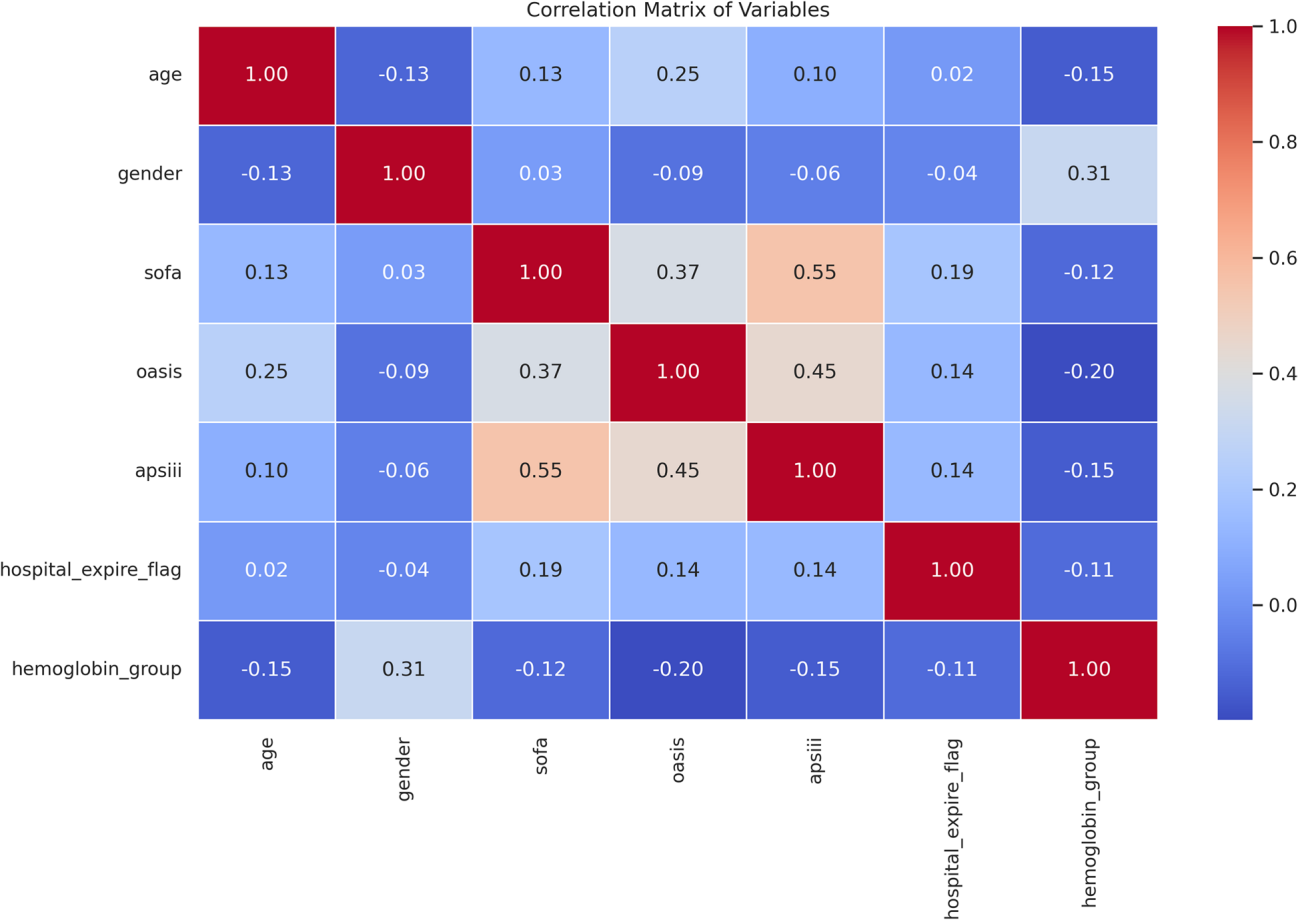
Heatmap of relevant variables

**Fig. 9:**
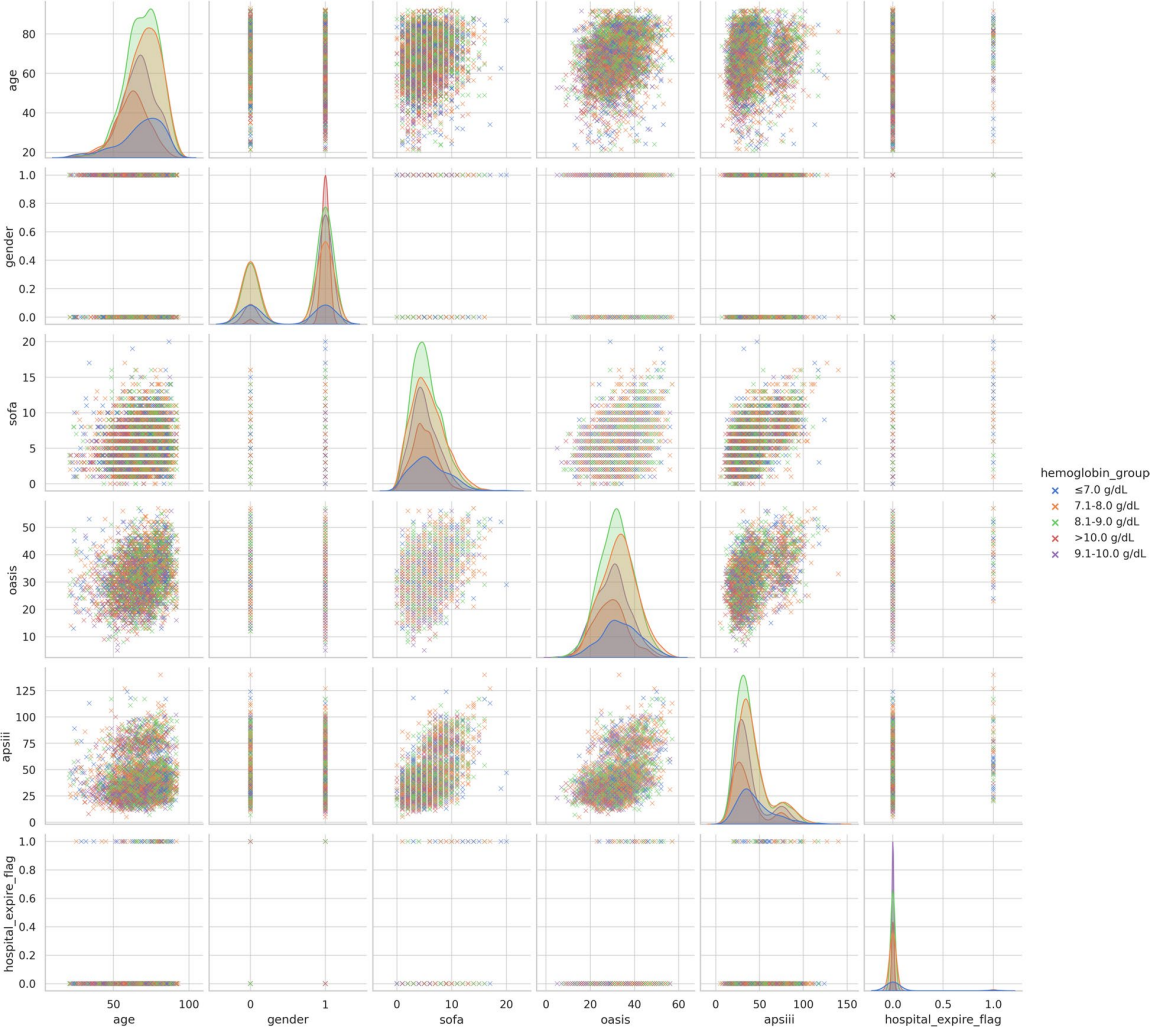
Matrix plot showing relationships between different variables

To further understand the relationship between hemoglobin levels and other relevant variables such as age, gender, in-hospital mortality, and various scores, we created a correlation heatmap (Fig. 8) and a matrix plot (Fig. 9). These figures show the degree of association between variables. The color intensity represents the strength of the correlation, and the size of the circles reflects the magnitude of the correlation.

#### Age and other variables

Age showed a mild negative correlation with gender and hemoglobin group, indicating that older patients might be more likely to have lower hemoglobin levels.

Age and SOFA, OASIS, and APSIII scores were positively correlated, suggesting that older patients might have higher disease severity.

#### Gender and other variables

Gender showed a moderate positive correlation with the hemoglobin group, indicating a certain association between gender and hemoglobin levels.

#### Relationship between scoring systems

SOFA, OASIS, and APSIII scores were positively correlated, suggesting that these scoring systems might share some common elements or factors when assessing disease severity.

Relationship between scoring systems and hemoglobin groups: All scoring systems showed negative correlations with hemoglobin groups, indicating that lower hemoglobin levels were associated with higher disease severity.

#### In-hospital mortality and other variables

In-hospital mortality showed a mild positive correlation with SOFA, OASIS, and APSIII scores, suggesting that higher scores might be associated with an increased risk of in-hospital mortality.

## Discussion

With the advancement of medical research, transfusion strategies for critically ill patients have become a critical part of clinical decision-making. Transfusion is a crucial method to adjust hemoglobin levels in patients, but it also comes with potential adverse reactions and complications. Excessive transfusion may lead to fluid overload, electrolyte imbalances, and immune response activation, increasing the length of hospital stay and medical costs. Therefore, determining the optimal transfusion strategy is essential to balance treatment efficacy and risk and improve patient outcomes [20].

In current clinical practice, it is generally recommended to consider transfusion when hemoglobin levels in critically ill patients drop to 7 g/dL or below. However, whether this threshold is applicable to patients undergoing extracorporeal circulation auxiliary to open heart surgery remains a topic of exploration. The process of open heart surgery with extracorporeal circulation itself may cause a significant decrease in hemoglobin levels, compounded by postoperative factors such as inflammation and blood loss, potentially leading to lower hemoglobin levels in these patients. As such, determining an appropriate hemoglobin transfusion threshold is particularly crucial for this patient population.

In this study, we conducted an in-depth analysis of a large sample from the MIMIC-IV database to explore the relationship between hemoglobin levels and clinical outcomes in patients after open heart surgery with extracorporeal circulation. Our findings supported the correlation between hemoglobin levels and patient mortality. Lower hemoglobin levels were associated with higher mortality rates. However, it is noteworthy that when hemoglobin levels exceeded 7 g/dL, there was no significant difference in mortality rates among the groups. This might resonate with the red blood cell transfusion thresholds mentioned in the clinical practice guidelines by Carson JL et al. [8]. This suggests that maintaining hemoglobin levels above 7 g/dL may be a more reasonable and safe transfusion strategy, avoiding the potential adverse effects of excessive transfusion.

Additionally, our research indicated that lower hemoglobin levels were correlated with extended hospital stays, ICU admission durations, and mechanical ventilation times. This observation is consistent with the study by Murphy GJ et al., which showed that a liberal transfusion strategy post-cardiac surgery might result in prolonged hospital stays and a heightened risk of complications compared to a restrictive approach [21]. Furthermore, we identified a significant correlation between hemoglobin levels and complication risks, norepinephrine dosage, and red blood cell transfusion volume. This aligns with the findings of Hajjar LA et al., who observed a similar relationship in patients post-cardiac surgery [22].

Upon evaluating the data, we discerned that a higher hemoglobin (Hb) level is inversely related to age. This observation underscores the tendency for younger patients to manifest elevated hemoglobin concentrations. Remarkably, within the cohort characterized by Hb levels exceeding 10.0 g/dl, the male demographic predominates, accounting for 97%. This pronounced male predominance may hint at an inherent physiological predisposition enabling males to sustain elevated Hb levels. Consequently, it intimates an augmented susceptibility among males compared to their female counterparts. A disparate distribution was evident in conditions like myocardial infarction, congestive heart failure, and cerebrovascular diseases when assessed across varied Hb strata. Such disparities insinuate potential associations between hemoglobin levels and the predisposition to specific foundational ailments. Moreover, universally lower scores in assessment tools like OASIS, SOFA, and APSIII in the higher Hb cohorts allude to a relatively benign clinical presentation in these individuals. Furthermore, vital parameters, namely respiratory rate, heart rate, mean arterial pressure, and oxygen saturation, showcased discernible variations across the Hb categories. Such variances are conceivably linked to the interplay between an individual’s cardiopulmonary function and their corresponding hemoglobin levels. In encapsulation, our investigative outcomes resonate with numerous extant scholarly articles, thereby amplifying the credibility and potential clinical ramifications of our findings.

### Potential mechanisms

One of the pivotal mechanisms underlying the relationship between hemoglobin levels and clinical outcomes is the capacity for oxygen delivery and utilization. Hemoglobin, being the primary transporter of oxygen in the blood, plays a crucial role in ensuring tissues receive adequate oxygen supply. Lower levels of hemoglobin could compromise this delivery, leading to tissue hypoxia. Tissue hypoxia, especially in vital organs such as the heart, brain, and kidneys, can result in impaired organ function and increased morbidity and mortality.

Another aspect to consider is the cardiovascular stress response, especially during surgeries assisted by extracorporeal circulation. The heart and vascular system are subjected to significant stress during such procedures. A diminished hemoglobin level might exacerbate this stress, potentially leading to heightened risks of complications.

Furthermore, while red blood cell transfusions can bolster the hemoglobin levels, they are not without risks. Transfusions can introduce risks such as transfusion reactions, infections, and inflammatory responses. These adverse effects of transfusions might, in some cases, outweigh the benefits, especially when transfusions are administered without clear indications. Karkouti K et al. discussed the interrelationship of preoperative anemia, intraoperative anemia, and red blood cell transfusion as potentially modifiable risk factors for acute kidney injury in cardiac surgery [23].

Other potential confounders that might influence the relationship between hemoglobin levels and clinical outcomes include the baseline health status of the patient, the complexity and complications of the surgery, and other treatment modalities employed during the care process.

### Clinical implications

#### The clinical implications of our findings stretch across various domains of cardiac patient management

Transfusion Decisions: Our study provides valuable insights that could guide clinicians in making more informed transfusion decisions. Specifically, when hemoglobin levels fall below 7 g/dL, consideration for transfusion might be warranted.

Preoperative Assessment and Treatment: For patients slated for cardiac surgeries, a preoperative assessment of hemoglobin levels could be integral. Identifying and potentially treating those with reduced hemoglobin levels might better prepare them for the surgery, ensuring they have the best chances for favorable outcomes.

Risk Prediction for Complications: Hemoglobin levels can be employed as a marker to predict the risk of complications, thereby guiding subsequent clinical decisions and patient management. This is especially crucial as patients with lower hemoglobin levels might be at heightened risk for prolonged hospital stays, increased ICU admission durations, and extended mechanical ventilation times.

Postoperative Care and Management: Post-surgery, patients exhibiting lower hemoglobin levels might necessitate more vigilant monitoring and care. Recognizing and addressing these levels promptly could potentially mitigate the risk of complications and improve patient outcomes.

Incorporating these findings into clinical practice could greatly enhance the quality of care provided to cardiac patients, ensuring they receive the most appropriate and effective interventions tailored to their individual needs.

### Limitations and future directions

Limitations:Data from MIMIC-IV 2.2 may not represent the broader population, introducing potential biases. Retrospective design may lead to recall bias and missing data affecting the outcomes. Unaccounted confounding factors could influence the results. Lack of randomization prevents establishing causal relationships.

Future Directions: Conduct prospective randomized controlled trials to establish causal relationships. Explore similar studies in diverse populations to validate generalizability. Investigate biological and physiological mechanisms underlying the hemoglobin-outcome relationship. Design and test targeted intervention strategies to improve patient outcomes after open heart surgery.

## Conclusion

The study findings demonstrate that when hemoglobin levels are below 7 g/dl, there is an increase in mortality. However, when hemoglobin levels are above 7 g/dl, there is no significant change in mortality, providing support for guidelines that set transfusion alerts at this threshold. Nevertheless, there is a need for further exploration of other clinical outcomes, including hospital stay duration, ICU stay duration, mechanical ventilation duration, transfusion volume, and norepinephrine usage, among others. In-depth research into these outcome measures will enable a comprehensive understanding of the impact of hemoglobin levels on patients. This understanding can lead to the development of more effective treatment strategies, ultimately enhancing patient outcomes and improving their quality of life. Therefore, future research should prioritize and explore these directions to gain more insights into this field.

## Data Availability

The original data of our study are available from the corresponding author upon reasonable request. Publicly available datasets were analyzed in this study. This data can be found here:https://physionet.org/content/mimiciv.
